# Inimitable Impacts of Ceramides on Lipid Rafts Formed in Artificial and Natural Cell Membranes

**DOI:** 10.3390/membranes12080727

**Published:** 2022-07-23

**Authors:** Masanao Kinoshita, Nobuaki Matsumori

**Affiliations:** Department of Chemistry, Graduate School of Science, Kyushu University, Fukuoka 819-0395, Japan; matsmori@chem.kyushu-univ.jp

**Keywords:** lipid rafts, signal platforms, apoptosis, phase separation, lipid membranes, transmembrane signalling

## Abstract

Ceramide is the simplest precursor of sphingolipids and is involved in a variety of biological functions ranging from apoptosis to the immune responses. Although ceramide is a minor constituent of plasma membranes, it drastically increases upon cellular stimulation. However, the mechanistic link between ceramide generation and signal transduction remains unknown. To address this issue, the effect of ceramide on phospholipid membranes has been examined in numerous studies. One of the most remarkable findings of these studies is that ceramide induces the coalescence of membrane domains termed lipid rafts. Thus, it has been hypothesised that ceramide exerts its biological activity through the structural alteration of lipid rafts. In the present article, we first discuss the characteristic hydrogen bond functionality of ceramides. Then, we showed the impact of ceramide on the structures of artificial and cell membranes, including the coalescence of the pre-existing lipid raft into a large patch called a signal platform. Moreover, we proposed a possible structure of the signal platform, in which sphingomyelin/cholesterol-rich and sphingomyelin/ceramide-rich domains coexist. This structure is considered to be beneficial because membrane proteins and their inhibitors are separately compartmentalised in those domains. Considering the fact that ceramide/cholesterol content regulates the miscibility of those two domains in model membranes, the association and dissociation of membrane proteins and their inhibitors might be controlled by the contents of ceramide and cholesterol in the signal platform.

## 1. Introduction

Ceramide, which consists of a sphingosine base and an *N*-linked acyl-chain, is a key intermediate in the biosynthesis of complex sphingolipids, such as sphingomyelins, and glycosphingolipids, among others [1,2,3,4,5,6]. In the mammalian lipidome, there are many types of ceramides differing in their acyl chain and sphingosine backbone. The acyl chain length of physiologically relevant ceramides ranges from C14 to C27 carbon chains [7], which are largely saturated, while their sphingosine consists of C16–C20 carbon chains, which usually bear a trans-double bound linkage at the C5–C6 position [8]. Although ceramide is an extremely minor constituent of plasma membranes, it is drastically increased in stimulated cells [9,10,11,12,13,14]. Interestingly, such an increase in ceramide levels triggers basic cellular processes such as apoptosis [12,13,14], cell growth, differentiation [15], cycle arrest [16], and cellular senescence [17]. 

In the last two decades, artificial membrane studies have disclosed that the rigid and compact ceramide acts as a lipid packing modulator, altering the structure and physicochemical properties of phospholipid membranes. In such studies, one of the most remarkable findings is that ceramide causes structural alterations of lipid rafts [18,19,20]. Lipid rafts are sphingomyelin (SM)/cholesterol (Chol)-rich ordered membrane domains, in which signalling molecules and their receptors are abundantly incorporated [21,22,23,24,25]. Thus, lipid rafts have attracted multi-disciplinary interests due to their potential involvement in transmembrane signalling. Plasma-membrane ceramides are often generated in lipid rafts upon the activation of SMase, which is responsible for breaking SM down into a ceramide and a phosphorylcholine (PC) [22,23]. Thus, it is not farfetched to state that ceramide converted from SM in lipid rafts exerts its biological activity by alternating the structure of lipid rafts.

Following the theme of this special issue of *Membranes*, “artificial membranes and their applications”, we first discuss the characteristic properties of ceramide molecules and their capability to form domains in phospholipid membranes. Next, we show the impact of ceramide on lipid rafts formed in artificial and cell membranes along with some devised methodologies for observing the partition behaviour of natural ceramide. Finally, we discuss the influence of ceramide-induced structural alterations to lipid rafts on the regulation of transmembrane signalling.

## 2. Characteristic Properties of Ceramides and Their Domain Formation in Phospholipid Membranes

### 2.1. Characteristic Properties of Ceramide Molecules

**Figure 1:**
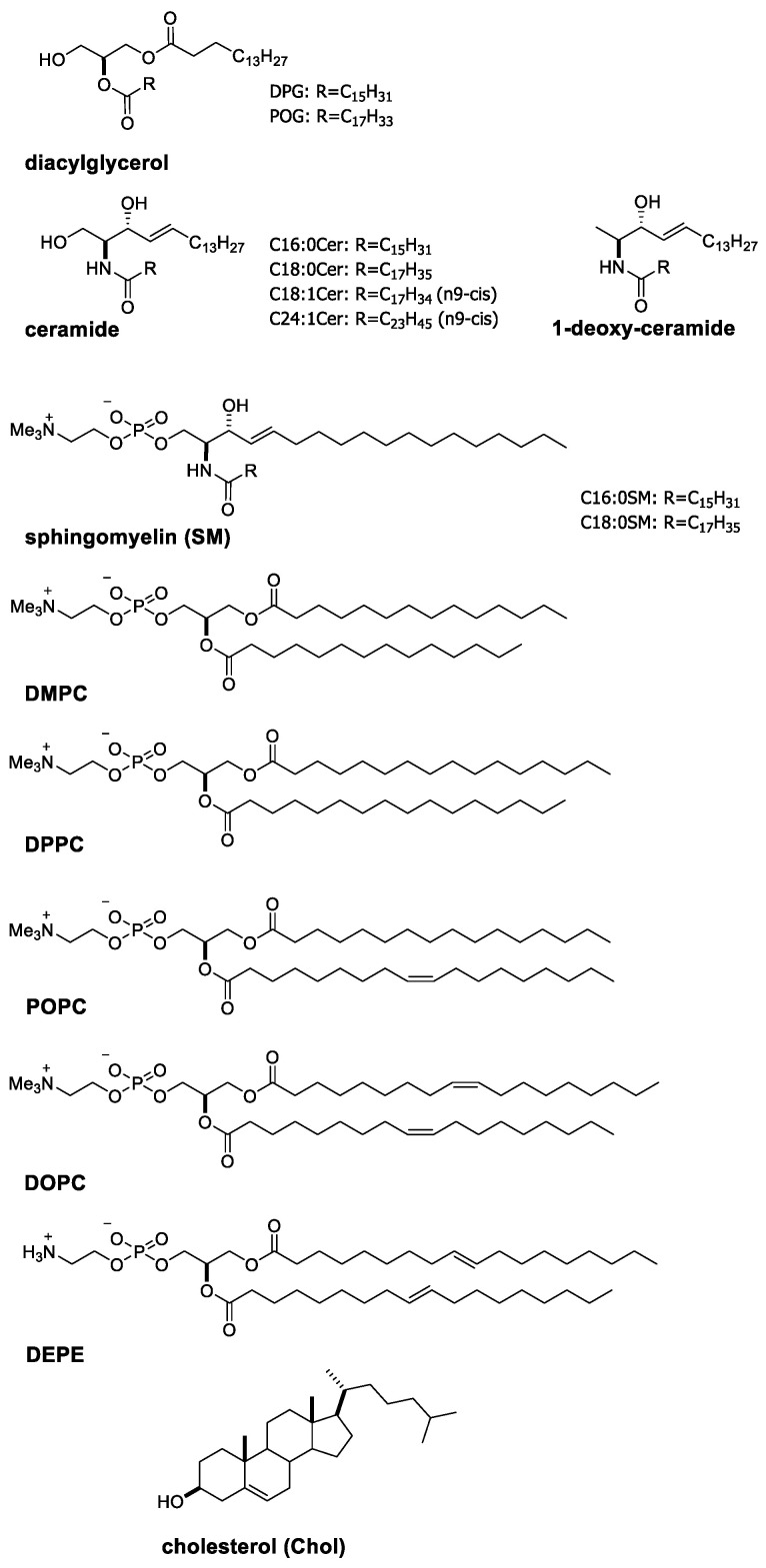
Chemical structures of ceramides, diacylglycerols, phospholipids and cholesterol described in the present article.

A characteristic property of ceramide is becoming evident when comparing it with diacylglycerol, which is a glycerol analogue of ceramide (chemical structures of the lipids described in the present article are shown in Figure 1). Differential scanning calorimetry (DSC) and temperature scanning Fourier-transform infrared spectroscopy (FTIR) measurements demonstrated that physiologically abundant palmitoyl-ceramide (C16:0Cer) membranes give rise to a chain melting transition at *T*_m_ ≈ 90 °C (Δ*H* = 56.5 kJ/mol) [26,27]. This *T*_m_-value is much higher than that of diacylglycerol membranes with corresponding acyl chains (dipalmitoylglycerol; DPG) (*T*_m_ = 62.5 °C) [28]. Moreover, a higher *T*_m_-value of the ceramide membrane was observed even when the palmitoyl acyl chain was substituted by an unsaturated chain; i.e., a *T*_m_-value of the oleoyl-ceramide (C18:1Cer) membrane (49 °C) was higher than that of the 1-palmitoyl-2-oleoyle-glycerol (POG) membrane (12 °C) [29,30].

A question at this stage is why ceramide membranes exhibit the higher *T*_m_-values than the diacylglycerol counterparts. As shown in Figure 1, ceramide possesses more abundant polar moieties, including two hydroxy and one amide groups at their interfacial regions in comparison with diacylglycerol, which possesses only a single hydroxy group. These polar moieties of ceramide can donate and accept (often water-mediated) hydrogen bonds with those of neighbouring ceramides [19,31,32,33,34]. In fact, the 1-hydroxy group of ceramide contributes to the intermolecular interaction because 1-deoxy-ceramide (C16:0) membranes (Figure 1) display 13 °C lower *T*_m_-values than normal C16:0Cer membranes [35]. In addition, FTIR experiments disclosed that the amide group of ceramide is also involved in the formation of a hydrogen bond network at the interface of ceramide membranes [36,37]. Furthermore, such intermolecular hydrogen bonds have been confirmed by a number of simulation studies [38,39,40]. Thus, the hydrogen bond capability, as a characteristic propensity of ceramide, confers the high thermal stability of ceramide membranes.

### 2.2. Formation of Ceramide-Rich Gel Domains in Phospholipid Membranes

**Figure 2:**
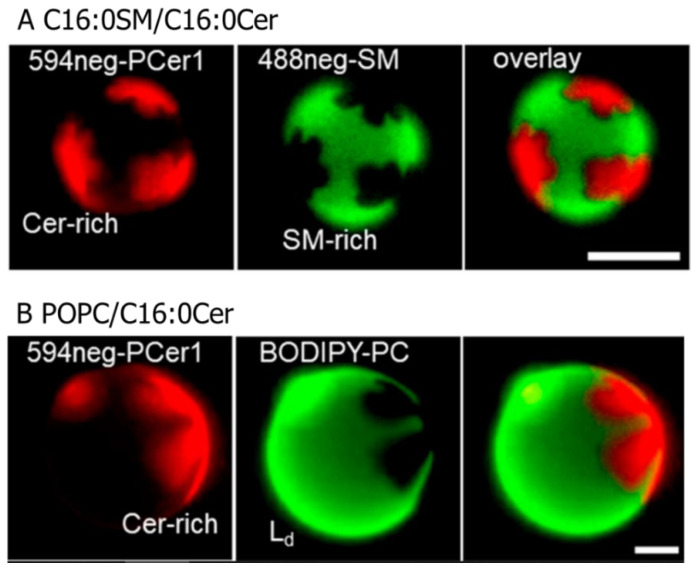
Fluorescence micrographs of binary-component giant unilamellar vesicles (GUVs) that underwent phase separation between the ceramide-rich and ceramide-poor (thus, phospholipid-rich) domains. (**A**) C16:0SM/C16:0Cer (95:5, molar ratio) GUVs containing 0.2 mol % 594neg-PCer1 (ceramide-rich domain marker) and 0.2 mol % 488neg-SM (SM-rich domain marker). (**B**) Palmitoyl-oleoyl–phosphatidycholine (POPC)/C16:0Cer (95:5, mole ratio) GUVs containing 0.2 mol % 594neg-PCer1 and BODIPY-PC (POPC-rich fluid phase marker). Bars indicate 10 μm. Image brightness and contrast were adjusted for clarity. This figure was adapted with permission from [41]. Copyright 2019 American Chemical Society.

Intermolecular interactions between ceramides promote their condensation, generating ceramide-rich gel-like domains in phospholipid membranes (Figure 2) [41]. Previously, researchers investigated the compositional dependence of the ceramide-rich domain formation in palmitoyl–oleoyl–phosphatidylcholine (POPC) bilayers (fluid phase). In that study, they added trans-parinaric acid (tPA), whose fluorescent half-life depends on membrane rigidity, to POPC/C16:0Cer binary bilayers and followed the appearance of the C16:0Cer-rich domains. It was disclosed that the formation of C16:0Cer-rich domains commenced at an extremely small molar fraction of C16:0Cer (*x*_Cer_ < 0.05) [42,43]. The ceramide-rich domains were formed even in gel membranes such as SM, dielaydoylphosphatidylethanolamine (DEPE), dipalmitoylphosphatidylcholine (DPPC), and dimyristorylphosphatidylcholine (DMPC) bilayers. In addition, the *T*_m_ of the ceramide-rich domain was shown to be higher than that of host membranes [44,45,46]. Moreover, surface pressure vs. molecular area isotherm measurements of sphingomyelin/ceramide monolayers revealed that the ceramide-rich domains show a significantly higher shear viscosity than the SM-rich (thus, ceramide-poor) domains [47,48,49,50]. These results indicate that the ceramide-rich domains are more ordered than the host membranes. It is likely that the intermolecular hydrogen bonding between ceramide–ceramide and/or ceramide-adjacent phospholipids enhances the order of the ceramide-rich domains, conferring the high shear viscosity and *T*_m_-values to the ceramide-rich domains. Moreover, Pinto et al. compared the domain-formation capability of ceramides with saturated (C16:0, C18:0, and C24:0) and unsaturated (C18:1 and C24:1) acyl chains in POPC bilayers. While saturated ceramides begin to form the ceramide-rich domains with only a small molar fraction of ceramides (*x*_Cer_ < 0.05), relatively large amounts of unsaturated ceramides are required for their domain formation (*x*_Cer_ ≈ 0.2) [43]. It is likely that the kinked conformation of the unsaturated carbon chain prohibits close contact between ceramide molecules and, thus, attenuates intermolecular hydrogen bonding.

The capability of ceramides for their domain formation is often compared with that of Chol because it also forms Chol-rich domains termed the liquid-ordered (Lo) phase in phospholipid bilayers [51,52,53]. Previously, we investigated the chain-packing structure of the ceramide-rich domain formed in C16:0SM/C16:0Cer mixtures using wide-angle X-ray diffraction (WAXD) [54]. This experiment showed that the ceramide-rich domain gives a sharp WAXD peak at ≈2.37 nm^−1^, demonstrating that the carbon chains form a hexagonal packing with the lattice spacing of 0.42 nm (=1/2.37 nm). This WAXD pattern is similar to that of the gel phase formed by usual phospholipid bilayers. In contrast, the Chol-rich Lo domains gave a broad WAXD peak at a smaller angular region (≈2.1 nm^−1^) [55], indicating that the chain packing of the Lo domains is looser than that of the ceramide-rich gel domains. Moreover, deuterium nuclear magnetic resonance (^2^H NMR) measurements showed that the addition of ceramide increases the order of the chain packing in C16:0SM/Chol bilayers (homogeneous Lo phase) [56]. Thus, ceramide is superior to Chol in terms of membrane-ordering effects. This further suggests that the ceramide concentration has to be kept at a very low level in normal cell membranes to prevent the formation of the gel phase, which is not a preferred structure in fluid biomembranes.

### 2.3. Ceramide-Induced Fusion of Raft-like Domains

**Figure 3:**
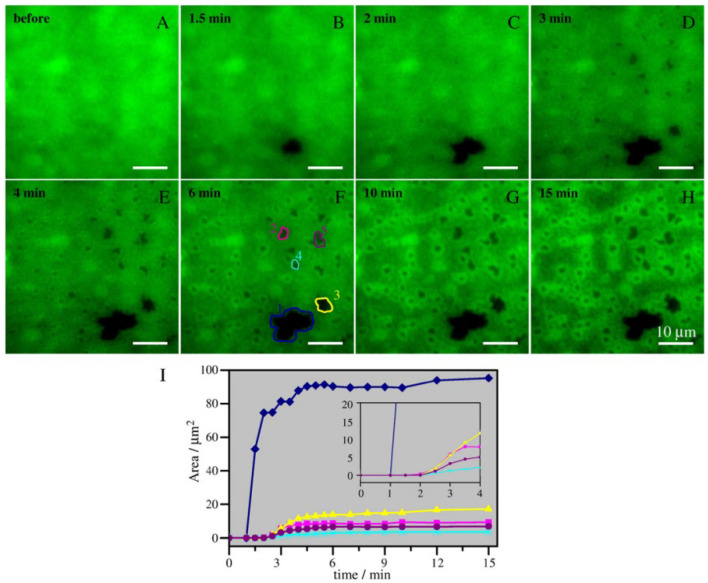
Real-time sphingomyelinase (SMase)-induced coalescence of raft-like ordered domains visualised using total internal reflection fluorescence (TIRF) microscopy. Egg-SM/Chol/dioleoylphosphatidylcholine (DOPC) (5:1:5 in molar ratio) bilayers, which contained 0.5% fluorescently labelled dipalmitoylphosphatidylethanolamine (a non-raft domain marker), were imaged before (**A**) and at various times after (**B**–**H**) injection of 1 U/mL SMase. The dark regions correspond to raft-like domains. (**I**) Areas of “domains” 1–5 (outlined in panel F) were plotted as a function of time after enzyme injection; the inset shows initial slopes of the curves. The graphs show expansion of the dark patches during the first 0–15 min. This figure was redrawn from [58] with permission from Elsevier (License No. 5334110330270).

It has been reported that physiologically abundant ceramides, such as palmitoyl and stearoyl ceramides (C16:0Cer and C18:0Cer, respectively) are preferentially incorporated into raft-like ordered domains, leading to fusion of the raft-like domains. Murthy et al. prepared milk-SM (main acyl chain; C23:0)/dioleoylphosphatidylcholine (DOPC)-supported bilayers, in which SM-rich raft-like domains were phase-separated from the DOPC-rich fluid matrix. They found that the addition of C16:0Cer to the sample resulted in enlargement of the SM-rich domains [57]. Ira and Johnston examined the influence of in situ ceramide generation on egg-SM/Chol/DOPC (5:1:5 molar ratio) supported bilayers [58]. In this mixture, egg-SM/Chol-rich nanodomains, whose size (tens to a few hundred nanometres) is close to that of lipid rafts formed in cell membranes, are randomly dispersed in DOPC-rich fluid matrix [58]. Their time-lapse fluorescence observation revealed that the generation of ceramides by SMase promotes clustering of the nanodomains into large patches (Figure 3). The authors speculate that mechanical stress generated by the SM-to-ceramide conversion leads to the fusion of pre-existing egg-SM/Chol-rich domains because the domain fusion was not observed when the ceramide was pre-mixed with the lipid composition to reduce mechanical stress [58]. However, such fusion of the raft-like domains depends on the lipid composition because the ceramide-induced domains fusion was not observed when the SM/Chol/POPC mixture was used instead of the SM/Chol/DOPC mixture [59].

**Figure 4:**
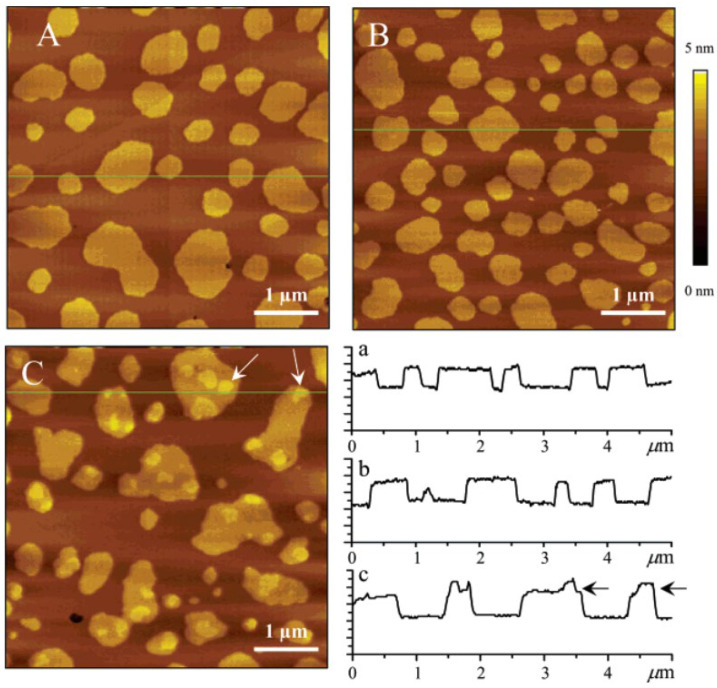
AFM images of C16:0SM/Chol/DOPC (2:1:2 molar ratio) bilayers containing 0 (**A**), 5 (**B**), and 10 (**C**) mol % of C16:0Cer. The total mole percentage of SM plus ceramide was kept constant at 40%. All images are 5 × 5 µm with a z-scale of 5 nm. Cross-sections for lines indicated in images (**A**–**C**) are shown in a, b, and c, respectively. The Lo domains showed higher membrane thickness while the Ld domains showed lower. Thus, the brighter and darker regions correspond to the Lo and Ld domains, respectively, in these AFM images. Some examples of the convex subdomains for 10% ceramide are indicated by arrows in image C and cross-section c. This figure was adapted with permission from [61]. Copyright 2006 American Chemical Society.

Furthermore, some investigators have addressed distribution of ceramide inside the raft-like domains. Sot et al. observed ceramide distributions in the free-standing giant unilamellar vesicles (GUVs) consisting of egg-SM/Chol/egg-PC/egg-PE/egg-ceramide, which undergo macroscopic phase separation between the raft-like liquid ordered (Lo) and non-raft-like liquid disordered (Ld) domains [60]. In this experiment, they decorated GUV samples with fluorescently labelled ceramide (7-nitrobenz-2-oxa-1,3-diazol-4-yl-ceramide; NBD-Cer). While NBD-Cer does not exhibit the same partitioning behaviour as natural ceramide, it is preferentially recruited in both Lo and Ld domains but is excluded from the gel phase [60]. As a result, the authors found that gel-like dark domains are formed inside the Lo phase. Moreover, a number of groups have observed the membrane thickness of the Lo phase formed in SM/Chol/DOPC/ceramide-supported bilayers using atomic force microscopy (AFM) [61,62]. They discovered that some convex subdomains are formed inside the Lo domain in the ceramide-containing sample, whereas a smooth Lo surface was observed in the ceramide-free sample (Figure 4). The formation of the subdomains inside the Lo phase was observed even in the case of in situ ceramide generation by SMase in egg-SM/Chol/DOPC (2:1:2 by moles)-supported bilayers [61,63]. Since the size and number of subdomains increase as the ceramide content increases, the subdomains are likely enriched in ceramide. However, direct evidence for this has not been obtained because AFM and fluorescent observation of NBD-Cer cannot access the distribution of natural ceramide.

### 2.4. Devised Methodologies for Visualising Ceramide Distribution

**Figure 5:**
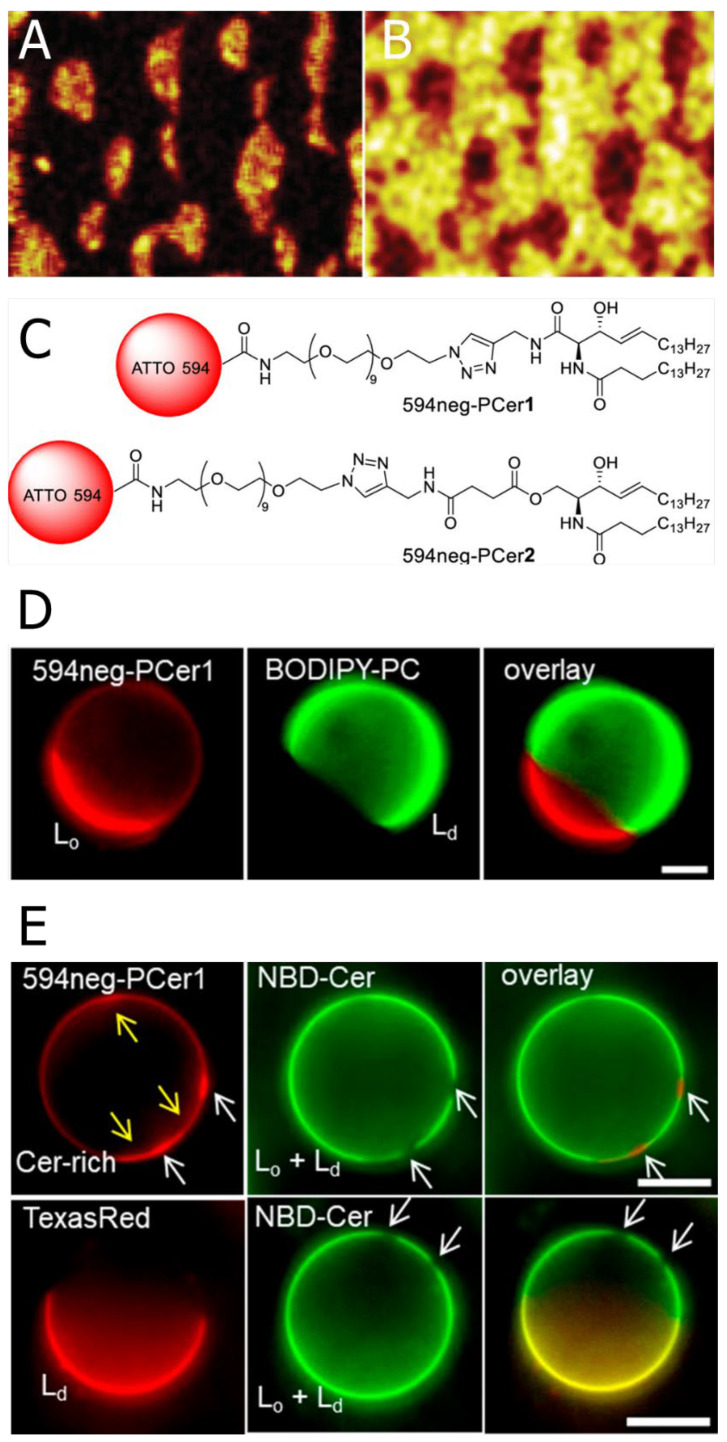
(**A**,**B**) Time-of-flight secondary ion mass spectrometry (ToF-SIMS) images for C16:0SM/Chol/DOPC/C16:0Cer-d31 (3:2:4:1 molar ratio) monolayers deposited on the substrate at 30 mN/m. (**A**) shows distribution of m/z 2 signals due to deuterium in the negative ion mode that is used to monitor the location of deuterated ceramide. (**B**) shows distribution of m/z 281 signals due to the oleate fragment that was used to monitor DOPC. (**C**) illustrates chemical structures of newly developed fluorescent ceramide analogues 594neg-PCer1 and 594neg-PCer2 (inclusively termed 594neg-PCer). (**D**) Fluorescence micrographs of C16:0SM/Chol/DOPC (1:1:1 molar ratio) ternary component GUVs that underwent phase separation between the Lo and Ld domains. This sample contained 0.2 mol % 594neg-PCer1 and BODIPY-PC (Ld marker). (**E**) Fluorescence micrographs of C16:0SM/Chol/DOPC/C16:0Cer (1:1:1:0.3 mole ratio) quaternary component GUVs. This sample contained (top) 0.2 mol % 594neg-PCer1 and 0.2 mol % NBD-Cer (both Lo and Ld marker) and (bottom) 0.2 mol % Texas Red-DPPE (Ld marker) and 0.2 mol % NBD-Cer. White and yellow arrows indicate the ceramide-rich subdomains and Lo domains, respectively. Bars indicate 10 μm. This figure was adapted with permission from [41,65]. Copyright 2008 and 2019 American Chemical Society.

Since the partition behaviour of natural ceramides is altered often by fluorescent labelling (Figure 5E), direct observation of the ceramide distribution is a long-standing issue [64]. Here, we showed some devised methodologies for visualising ceramide distribution in lipid membranes.

Popov et al. observed the distribution of C16:0Cer in an SM/Chol/DOPC/C16:0Cer-d31 (4:3:2:1 molar ratio)-supported monolayer using time-of-flight secondary ion mass spectrometry (Tof-SIMS) imaging [65]. This mass spectrometry-based imaging allows the chemical mapping of each lipid in multi-component membranes [65,66,67,68,69,70]. In this experiment, the authors used lipids with fully deuterated acyl chains, which provide a characteristic signal that traces specific lipids. Using this methodology, they visualised the heterogeneous distribution of ceramide (thus, the formation of the ceramide-rich subdomains) inside the SM/Chol-rich Lo phase (Figure 5A,B). However, because Tof-SIMS observation requires dried and fixed samples, it is of some concern that membrane structures may be altered in the process of sample preparation. 

Our group developed new fluorescent ceramide analogues: 594neg-PCer1 and 594neg-PCer2 (inclusively termed 594neg-PCer; Figure 5C). Unlike conventional fluorescently labelled ceramides, which bear a hydrophobic fluorophore on their acyl chains, 594neg-PCer conjugates hydrophilic fluorophores such as ATTO594 to the ceramide headgroup via a hydrophilic nona-ethylene glycol (neg) linker. This design places the fluorophore (ATTO594) some distance away from the membrane surface and, thus, greatly reduces its perturbing effect on membranes [71]. Therefore, 594neg-PCer displays a similar distribution to natural ceramides. In fact, we confirmed that 594neg-PCer is preferentially recruited in the ceramide-rich domains formed in phospholipid/ceramide binary bilayers (see Section 2.2 and Figure 2 for the phase behaviour of phospholipid/ceramide bilayers and distribution behaviour of 594neg-PCer, respectively). Using this ceramide probe, we observed the distribution of 594neg-PCer in Lo/Ld phase-separated GUVs consisting of C16:0SM/Chol/DOPC (1:1:1 molar ratio) and found that 594neg-PCer was preferentially recruited in the Lo phase (Figure 5D). However, in this sample, the ceramide-rich subdomains were not observed inside the Lo domain, which was probably because the concentration of the 594neg-PCer (0.2 mol % of total lipids) was too low to construct the ceramide-rich subdomains. Thus, we added non-labelled ceramide to the sample and observed distribution of the 594neg-PCer in C16:0SM/Chol/DOPC/C16:0Cer (1:1:1:0.3 molar ratio) GUVs. Consequently, we observed local condensation of the 594neg-PCer (indicated by white arrows in Figure 5E top) inside the Lo phase (indicated by yellow arrows in Figure 5E top). Since 594neg-PCer shows the similar distribution to natural ceramide, this result manifests that ceramide molecules assemble to form the ceramide-rich subdomains inside the Lo phase. Although 594neg-PCer is useful for observing ceramide distributions on the membrane surface, it has not been confirmed that 594neg-PCer can trace the trafficking behaviour of natural ceramides between the plasma-membrane and organelles.

In addition, fluorescently-labelled anti-ceramide antibodies have been employed for observations of ceramide distribution, especially in cell membranes, providing direct evidence for the formation of ceramide-rich domains and their structural alteration upon cell stimulation (see the next section for details). Thus, there is no doubt that the anti-ceramide antibody has been a useful tool to visualise the ceramide distribution. However, it is of concern that binding of the extremely large antibody might unintentionally modify the intrinsic properties of native ceramides.

### 2.5. Ceramide-Induced Compositional Alteration of Raft-like Ordered Domains

Ceramide leads not only to structural alteration but also to compositional changes involving the raft-like Lo domains. In a pioneering study, Megha and London examined the influence of C18:0Cer on the lipid composition of dipalmitoylphosphatidylcholine (DPPC)/Chol-rich Lo domains formed in DPPC/Chol/DOPC (1:0.35:1 molar ratio) bilayers [72]. These samples contained radioactive and fluorescent analogues of Chol, ^3^H-Chol, and dehydroergosterol (DHE), respectively, to measure Chol content in the Lo domains. They discovered that the Chol content in the detergent-extracted Lo domains was significantly reduced in a ceramide-containing sample. Later, such Chol replacement by ceramide was confirmed by different approaches such as fluorescence measurements [73,74,75], calorimetry [76], and AFM [62]. Although a detailed mechanism is not known, we suppose that ceramide interacts with the Lo lipids more effectively than Chol dose because of the excellent hydrogen bond formability of ceramide (see Section 2.2). Thus, ceramide can be incorporated effectively into the Lo phase by replacing Chol.

Further detailed analysis of ceramide-induced compositional alterations of the Lo phase was conducted by Alonso’s group. González-Ramírezand et al. investigated the phase behaviour of C16:0SM/Chol/C16:0Cer (*x*:*y*:*y* molar ratio) bilayers using AFM and fluorescence measurements [77]. At low C16:0Cer/Chol-content (*y* < 0.2), C16:0SM/Chol/C16:0Cer membranes undergo phase separation between the Lo and gel domains, which are enriched in C16:0SM/Chol and C16:0SM/C16:0Cer, respectively. This result seems reasonable, because C16:0Cer replaces Chol in the Lo phase and, thus, a part of the SM/Chol-rich Lo domains transforms into the C16:0SM/C16:0Cer gel domains. However, at a higher C16:0Cer/Chol-content (*y* = 0.39), the authors observed an almost homogenous phase. This result is consistent with the calorimetric analysis by Busto et al. [78]. Their DSC measurements demonstrated that C16:0SM/30 mol % Chol bilayers (homogeneous Lo phase) showed a symmetric broad transition peak, whereas C16:0SM/C16:0Cer (*x*_Cer_ < 0.30) bilayers showed an asymmetric sharp transition peak. Hence, they expected that if C16:0Cer replaces Chol, the addition of C16:0Cer to the C16:0SM/30 mol % Chol bilayer would cause a transformation from symmetric to asymmetric transition peaks as a consequence of formation of the C16:0SM/C16:0Cer-rich domains. However, increasing the amount of C16:0Cer up to 30 mol % did not significantly change the peak shape, indicating that Chol is not replaced by C16:0Cer. However, this result does not necessarily indicate the exclusion of C16:0Cer from the Lo domains because the characteristic phase transition of pure C16:0Cer (*T*_m_ ~ 90°C), which should be formed as a consequence of the exclusion of C16:0Cer from the Lo phase (see Section 2.1), was not observed. Therefore, the authors inferred the formation of a C16:0SM/C16:0Cer/Chol three-lipid mixed phase at a high ceramide/Chol-content. This is probably because, at high ceramide/Chol content, ceramide–Chol interactions become more dominant than SM–ceramide and/or –Chol interactions [77]. Taken together, these results suggest that the ceramide-induced replacement of Chol depends on the ceramide/Chol content; namely, ceramide replaces Chol when the content of Chol/ceramide is less than 20 mol %, while a favourable mixing of ceramide and Chol is achieved at higher concentrations of ceramide/Chol [79]. 

## 3. Formation of Ceramide-Enriched Signal Platforms and Their Biological Functions

### 3.1. Ceramide-Induced Signal Platform Formation and Transmembrane Signalling

Ceramide is involved in a variety of biological functions, and its role in Fas-related apoptotic signalling has been widely investigated. The Fas receptor can express weak activity through antibody-mediated engagement, whereas the micro-sized cluster of Fas drastically amplifies its apoptosis signal [80]. Thus, some investigators hypothesised that the formation of microclusters is a prerequisite for the effective transmembrane signalling of Fas [81,82]. Although Fas molecules form clusters in a lipid raft-mediated manner, the putative size of such lipid rafts (several to tens of nanometres) in normal cell membranes is much smaller than that of Fas clusters (several hundred nanometres to several micrometres) [68,83,84,85,86]. Previously, Gulbins et al. reported that cytosolic SMase translocates rapidly from intracellular stores to lipid rafts immediately after cellular stimulation, leading to ceramide generation [87,88]. Following ceramide generation, the formation of micro-sized domains (termed signal platforms) was observed by fluorescence microscopy. Although the process of signal platform formation is not known [89], the generated ceramides likely cause the coalescence of pre-existing SM/Chol-rich lipid rafts into a large platform, as observed in artificial membranes (see Section 2.3). Consequently, Fas molecules are recruited to the signal platforms and form micro-clusters. This mechanism is supported by the fact that the clustering of Fas and its signal transduction are suppressed by the depletion of SMase and the neutralisation of ceramide with anti-ceramide antibodies [87,88]. This motif proposed by Gulbins’s group is probably not limited to Fas clustering because other membrane proteins such as glycophosphatidylinositol-anchored proteins (GPI-APs) [71,90], CD28 [91], CD40 [92], CD95 [87], T-cell receptors (TCRs) [93], and B-cell receptors (BCRs) [94] also form clusters in signal platforms.

### 3.2. A Possible Structure of Signal Platforms

Although the structure of signal platforms is insufficiently understood, some clues regarding their lipid composition have been obtained in previous studies. For example, Bionda et al. revealed that signal platforms are effectively dyed by fluorescently labelled lysenin [95], which is a representative SM-binding protein [96,97]. Dumitru et al. reported that methyl-*β*-cyclodextrin-induced Chol extraction from cell membranes abrogates the formation of signal platforms [98]. These results indicate that signal platforms contain SM and Chol to some extent, together with ceramide, which is generated by the activation of SMase. In this case, we infer that the SM/Chol-rich and SM/ceramide-rich regions are phase-separated inside a single signal platform because artificial membrane studies showed that SM/Chol/ceramide mixtures undergo phase separation between these two domains at low ceramide/Chol-contents (see Section 2.5). Such a phase separation inside a signal platform is considered to be beneficial for compartmentalising membrane proteins and their inhibitors in different domains. In fact, it was reported that the potassium channel Kv1.3 localises in SM/Chol-rich pre-existing lipid rafts [99], while Src-like tyrosine kinases such as Lck, which are responsible for phosphorylation and inhibition of the channel, are localised in ceramide-rich domains [82,100,101]. Here, the question arises as to how proteins and their inhibitors associate inside a signal platform. In accordance with model membrane studies, the miscibility between the SM/Chol-rich and SM/ceramide-rich phases depends on the contents of ceramide and Chol. Namely, the phase separation between SM/Chol-rich and SM/ceramide-rich phases was observed at a low ceramide/Chol-content, while an almost homogenous phase was observed at a high content (see Section 2.5). Therefore, we proposed that the ceramide/Chol-content in signal platforms regulates the miscibility between these two phases and, thus, the association of membrane proteins and their inhibitors. Conversely, lowering the ceramide/Chol content promotes the dissociation of membrane proteins and inhibitors. However, direct information regarding the structure of signal platforms has not been obtained; thus, further structural analysis is needed.

### 3.3. Promising Ceramide-Analogues for Identifying Intrinsic Ceramide Functions

**Figure 6:**
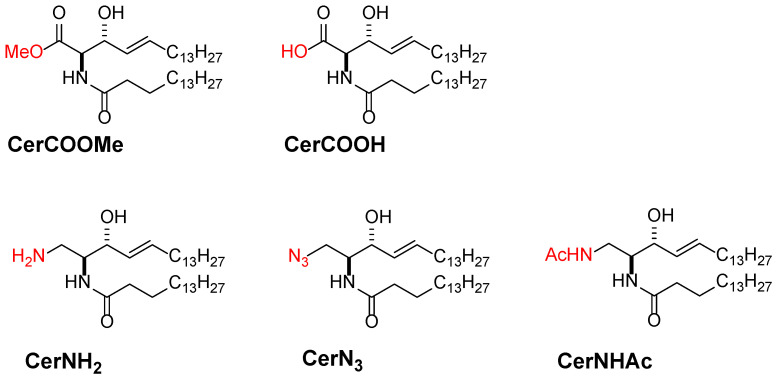
Chemical structures of 1-OH group substituted ceramide analogues developed in [102,103]. Protective groups are indicated by red colour.

While ceramide is central to signal transduction regulation, the biological functions of natural ceramides are poorly accessible because ceramide is readily transformed into more complex sphingolipids, such as SM and glycosphingolipids, via the modification of its 1-OH group [64,92]. To avoid this biosynthetic conversion, our group developed oxidised and nitrogen analogues of ceramides, in which the primary alcohol moiety was derivatised to -COOH, -COOMe, -NH_2_, -NHAc, and -N_3_ groups (Figure 6) [102,103]. In addition, we observed their capability of the domain formation in SM and POPC bilayers. Fortunately, these ceramide analogues retain their domain formability, at least to some extent. It is surprising that the -N_3_ analogue can form ceramide-rich domains, although it loses its hydrogen bond functionality. Thus, these ceramide analogues represent convenient tools for understanding ceramide-relevant biological functions because they are devoid of biosynthetic conversion. In particular, the -N_3_ analogue can be used for Raman imaging of ceramides because the -N_3_ group possesses striking Raman activity. Moreover, the -N_3_ and -NH_2_ groups are applicable for the in situ conjugation of fluorescent and other tags via Huisgen cycloaddition and condensation reactions, respectively. Hence, these ceramide analogues are promising molecular probes for understanding the mechanistic links between ceramide generation and biological function.

## 4. Summary

In the present review, we summarised the distribution of ceramides in phospholipid bilayers. It has been reported that an extremely low content of biologically relevant ceramides can form ceramide-rich domains because of their intermolecular hydrogen-bond capability. In addition, the ceramide-rich domains showed higher thermal stability (thus, a higher *T*_m_-value) and shear viscosity than host membranes consisting of diacyl-phosphatidylcholines and SMs. This indicates that ceramide has a particular propensity for membrane ordering. Moreover, artificial membrane studies disclosed that ceramide gives rise to a fusion of raft-like ordered membrane domains. Interestingly, such a ceramide-induced coalescence of pre-existing lipid rafts is also observed in real cell membranes; microscopic observations have shown the formation of micrometre-scale signal platforms immediately after cell stimulation and ceramide generation. Therefore, some investigators hypothesised that the membrane proteins are clustered in signal platforms, triggering transmembrane signalling. In addition, model membrane studies demonstrated that ceramide is preferentially recruited in the raft-like Lo phase, leading to the phase separation between the SM/Chol-rich and SM/ceramide-rich domains inside the Lo domain. Based on these results, we proposed that such phase separation occurs even in the signal platform formed in cell membranes. This structure of the signal platform is considered to be beneficial for the compartmentalisation of membrane proteins and their inhibitors in different regions. Moreover, according to model membrane studies, an increase in ceramide/Chol content promotes the miscibility of these two domains. Thus, the association and dissociation of membrane proteins and their inhibitors may be controlled by the ceramide/Chol-content in signal platforms.

Recently, ceramide has been piquing medical and pharmaceutical interests because ceramide could be a target molecule for cancer therapy [104,105,106]. It is widely known that the quantitative balance between ceramide and sphingosine-1-phosphate (S1P) (called sphingolipid rheostat) determines the cell fate, since ceramide and S1P mediate cell death and survive signals, respectively [107]. Interestingly, such a role of the sphingolipid rheostat works even in cancer cells, determining the initiation, progression and drug sensitivity of cancer cells [107,108,109]. Thus, regulation of the ceramide-induced signal transduction is essential for the clinical application of ceramide. However, certain issues still remain to be solved for understanding the mechanism of ceramide-related signal transduction. For example, the mechanism of ceramide-induced coalescence of lipid rafts remains unknown. In addition, ceramide-induced compositional alterations to lipid rafts and the underlying mechanisms have not been elucidated. Moreover, differences involving the physicochemical properties of lipid rafts and signal platforms are unknown. We expected that artificial membrane studies are anticipated to provide important insights into the functions and roles of ceramide in authentic cell membranes.

## Data Availability

Not applicable.
